# Correlation of the Expression Profile of Peripheral Leukocyte and Liver Tissue Immune Markers With Serum Liver Injury Indices in Children With Biliary Atresia

**DOI:** 10.1155/mi/9889239

**Published:** 2025-04-16

**Authors:** Anna Helmin-Basa, Izabela Kubiszewska, Joanna B. Trojanek, Małgorzata Wiese-Szadkowska, Maria Janowska, Zbigniew Kułaga, Joanna Pawłowska, Jacek Michałkiewicz

**Affiliations:** ^1^Department of Immunology, Collegium Medicum in Bydgoszcz, Nicolaus Copernicus University in Torun, Bydgoszcz, Poland; ^2^Department of Microbiology and Clinical Immunology, The Children's Memorial Health Institute, Warsaw, Poland; ^3^Department of Pediatric Surgery and Organ Transplantation, The Children's Memorial Health Institute, Warsaw, Poland; ^4^Department of Public Health, The Children's Memorial Health Institute, Warsaw, Poland; ^5^Department of Gastroenterology, Hepatology, Nutritional Disturbances and Pediatrics, The Children's Memorial Health Institute, Warsaw, Poland

**Keywords:** biliary atresia, gene expression, immune cells markers, liver injury indices, matrix metalloproteinases, tissue inhibitors of metalloproteinases

## Abstract

The aim of the study was to find associations between the levels of liver injury serum markers and the selected liver, peripheral leukocytes, and plasma immune characteristics in biliary atresia (BA) children. Twenty-five newly diagnosed BA children aged 4–30 weeks and 12 age-matched controls were included (for leukocytes characteristics) and 19 BA children and 11 controls (for liver studies). The frequencies of T helper 1 (Th1), Th2, Th17, Th17.1 cells as well as numbers of regulatory T (Treg), B cell subsets, and matrix metalloproteinase −2 and −9 (MMP-2 and MMP-9) expressing leukocytes in the whole blood were evaluated by flow cytometry. Plasma concentrations of tissue inhibitors of metalloproteinase (TIMP)−1, −2, MMP-9, interleukin-17A (IL-17A) and IL-6 were assessed by enzyme-linked immunosorbent assay (ELISA). The leukocyte and liver expression of the retinoic acid receptor-related orphan nuclear receptor gamma (*RORγT*), fork-head winged helix transcription factor P3 (*FoxP3*), transforming growth factor beta (*TGF-β*), interleukin-17A (*IL-17A*), *IL-6*, *IL-1β*, *IL-21*, interleukin 1 receptor antagonist (*IL-1Ra)*, *MMP-2*, *MMP-9*, *MMP-12* (liver only), *TIMP-1*, *TIMP-2*, T-box transcription factor expressed in T cells, also called TBX21 (*T-bet*), GATA-binding protein 3 (*GATA3*), and C-type lectin (*CD161*) mRNA were determined by real time RT-PCR (reverse-transcription polymerase chain reaction). The BA patients were characterized by increased frequencies of peripheral “suppressor” glycoprotein-A repetitions predominant protein (GARP)^+^latency-associated peptide (LAP)^+^Treg and activated Treg cells as well as MMP-2 and MMP-9 bearing lymphocytes, elevated plasma TIMP-1 levels, increased leukocyte expression of *MMP-9*, *TIMP-1*, *TIMP-2*, *IL-6*, and *TGF-β*, and decreased leukocyte expression of IL-21 and T-bet, increased liver expression of *FoxP3*, *TIMP-1*, and decreased liver expression of *IL-1β* and *MMP-2*. The following correlations were found between serum markers of liver injury and leukocyte and liver immune characteristics: (a) hemoglobin (Hb) levels correlated negatively with frequency of peripheral “suppressor” GARP^+^LAP^+^ Tregs; (b) aspartate aminotransferase (AST) levels correlated positively with frequency of the peripheral Th17.1 subset and expression of leukocyte *FoxP3*, (c) gamma glutamyltransferase (GGT) levels correlated positively with the peripheral memory B cells frequencies, the leukocyte *IL-6* and *TIMP-1* gene expression, (d) alanine aminotransferase (ALT) serum levels correlated positively with the naïve B cell frequency and liver *TIMP-2* expression, (e) total bilirubin (Bil) levels correlated positively with the leukocyte *MMP-9*, the plasma IL-6 levels, and the liver *TIMP-2* gene expression, (f) direct Bil levels positively correlated with the liver *IL-6* and *TIMP-2* expression, (g) international normalized ratio of prothrombin time (PT/INR) concentrations correlated positively with the peripheral Th17.1 subset frequency and the leukocyte *MMP-9* but negatively with the liver *FoxP3* expression. There were numerous strong positive correlations between the BA liver genes known to be involved in upregulation of IL-17 axis and MMPs/TIMPs expression. No prevailing leukocyte or liver single markers were uniquely associated with serum liver injury indices. BA immune profile is very complex with no single characteristics that would distinguish it from other liver inflammatory diseases.

## 1. Introduction

Biliary atresia (BA) is a progressive, highly inflammatory liver disease of largely unknown etiology that results in obliteration, fibrosis, and damage to the extrahepatic and intrahepatic bile ducts [1]. Inflammatory responses in BA, as in the other chronic liver diseases, result in progressive extracellular matrix (ECM) formation by activated hepatic stellate cells (HSCs). Enhanced HSC activation/proliferation by activated immune cells results in fibrosis, which can be inhibited either by HSC suppression or ECM degradation by matrix metalloproteinases (MMPs) [2]. Fibrosis in BA is enhanced by proinflammatory cytokines, mainly interferon gamma (IFN-*γ*), which promote HSC proliferation and secretion of fibrotic factors, including MMPs, tissue inhibitors of metalloproteinases (TIMPs), and collagen regulatory T cells (Tregs) inhibit T helper 1 (Th1) and/or IFN-*γ*-induced effects on HSCs by suppressing Th1-induced activation of signal transducer and activator of transcription 1 (STAT1). Intrahepatic IFN-*γ*/STAT1 levels increase along with the severity of fibrosis resulting from an imbalance between MMPs and TIMPs expression in the liver [3].

Relationships between BA clinical outcome and expression of immune markers in the liver and periphery, which would eventually be used as prognostic/diagnostic tools, have also been suggested, including (1) elevated plasma TIMP-1 levels associated with jaundice and portal hypertension [4], (2) increased population of activated peripheral blood natural killer cells (NK) cells along with elevated plasma interleukin-8 (IL-8) levels and decreased Treg population [5], (3) increased hepatic gene expression for Th1 cells [6], (4) increased number of intrahepatic CD4+ Th17 cells and elevated plasma levels of Th17-associated cytokines [7], and (5) changes in expression of MMPs and TIMPs in the liver and periphery associated with progression of fibrosis in different stages of BA [8], especially in the case of MMP-7, considered as a promising BA-specific biomarker [9], but recently questioned by others [10].

The aim of the present study was to evaluate the BA children immune profiles of plasma, leukocytes, and liver tissue and to determine which of them are associated with expression of the serum markers of liver injury. We evaluated the expression of some immune markers not previously studied in the BA children, including leukocyte expression of the MMP-2, MMP-9, TIMP-1, and TIMP-2, frequencies of activated, “suppressor” T cells bearing the glycoprotein-A repetitions predominant protein (GARP)+latency-associated peptide (LAP)+ phenotype, and distribution pattern of the pathogenic Th17.1 and B cell subsets.

## 2. Materials and Methods

### 2.1. Patients

The present prospective, single-center study was approved of the Ethical Committee at The Children's Memorial Health Institute (resolution no. 17/KBE/2017 of April 19, 2017) following “The Code of Ethics of the World Medical Association” (Declaration of Helsinki). Informed consent was obtained from parents/guardians after being fully informed about all study procedures before enrolment. All of the enrolled children underwent a complete history taking and clinical examination with a focus on clinical presentations.

All children were under care of the Department of Gastroenterology, Hepatology, Nutritional Disorders, and Pediatrics of The Children's Memorial Health Institute in Warsaw, Poland, from 2014 to 2019. The participants included 25 children with newly diagnosed BA aged 4–30 weeks, and 12 age-matched controls (for leukocyte phenotype characteristics). The BA children who underwent Kasai portoenterostomy (KPE) were prospectively recruited. The inclusion and exclusion criteria included complete obliteration of the bile ducts and the presence of biliary atresia splenic malformation (BASM), respectively. The final diagnosis was made on the basis of intraoperative evaluation of the biliary tract and cholangiography. Liver tissue specimens used for the gene expression analysis were obtained by the surgical liver biopsies during KPE procedure in 19 patients. Assessment of gallbladder, the presence of bile duct cysts, and the additional congenital defects were evaluated by means of abdominal ultrasound.

Liver biochemical serum parameters included: total and direct bilirubin (Bil), alanine aminotransferase (ALT), aspartate aminotransferase (AST), gamma-glutamyltransferase (GGT), prothrombin time (PT), and international normalized ratio (INR) (INR of PT [PT/INR]) at the time of diagnosis were available from the prospectively maintained database (Table 1).

Since liver biopsy could not be performed in the healthy children, the control group used for this study was recruited of the children suffering from cholestasis of unknown origin (*n* = 5) and bile duct cysts (*n* = 6) but without BA.

### 2.2. Flow Cytometry

Peripheral blood was collected in a TransFix/EDTA vacuum tube (Cytomark, Buckingham, UK). The active components of TransFix stabilize leukocytes and leukocyte antigens for up to 14 days. Blood was stored at 4°C until cytometry assays were performed. Flow cytometry experiments were performed on a FACS Canto II system (Becton Dickinson, San Diego, CA, USA) and analyzed using FlowJo software 7.5.5 (Tree Star, Inc. Ashland, Oregon, USA). Surface and intracellular staining of cells was performed for 30 min at room temperature (RT) or 4°C with fluorophore-labeled antibodies as listed in Table 2. Fluorescence minus one or isotype-matched antibodies were used as controls. For intracellular staining, the Cytofix/Cytoperm Fixation/Permeabilization Kit was used according to the manufacturer's instructions (BD Biosciences, San Jose, CA, USA). The expression of B cell subsets, Treg subsets, recent thymic emigrants (RTEs), selected Th subsets and MMPs in peripheral blood leukocytes was evaluated based on a multistep cell gating (Figures S1–S5).

### 2.3. RNA Isolation and Real-Time PCR Technique

The real-time technique used here has been described in detail previously [11]. Briefly, peripheral blood leukocytes were isolated from 4.9 ml of venous blood by Histopaque (Sigma-Aldrich 1119; Saint Louis, MO, USA) gradient centrifugation. Liver tissue specimens were obtained by the surgical liver biopsies. Total RNA was isolated in both leukocyte (mixed up and down) and liver tissue (minced on ice) by Chomczyński method using Trizol Reagent (Ambion; Carlsbad, CA, USA). Subsequently, the concentration and purity/integrity of RNA were determined by measuring absorbance at 260/280 nm. One microgram of total RNA per sample was converted to cDNA by real time RT-PCR (reverse-transcription polymerase chain reaction) using TaqMan reverse transcription reagents. Quantitative PCR (real-time PCR) was performed for the following target genes: retinoic acid receptor-related orphan nuclear receptor gamma (ROR*γ*T), fork-head winged helix transcription factor P3 (FoxP3), transforming growth factor beta (TGF-*β*), interleukin-17A (IL-17A), IL-6, IL-1*β*, IL-21, interleukin 1 receptor antagonist (IL-Ra), MMP-2, MMP-9, TIMP-1, TIMP-2, T-box transcription factor expressed in T cells, also called TBX21 (T-bet), GATA binding protein 3 (GATA3), C-type lectin (CD161), and endogenous control G3PDH on Viia 7 Real-Time System according to the manufacturer's recommendation. For one reaction, 50 ng of cDNA was applied with SYBR Green PCR Master Mix and 10 nmol/l for each forward and reverse primer (Table 3). Each sample was analyzed at least in duplicate. The specificity of the amplification reaction was verified by analysis of the melting curve, which was performed after each run. Relative fold changes in the mRNA level of target gene expression between the children with BA and the control group were determined by normalization to the mRNA expression of the reference gene G3PDH using Pfaffl's mathematical model [12]. All reagents, equipment, and other supplies for the real-time PCR technique were provided by Applied Biosystems (Thermo Fisher Scientific).

### 2.4. Cytokine Measurements

Plasma concentrations of MMP-9 and TIMP-1, TIMP-2, and IL-17A were determined using DuoSet enzyme-linked immunosorbent assay (ELISA) kits (R&D Systems; Minneapolis, MN, USA). IL-6 was determined using the OptEIA Set ELISA kits (Becton Dickinson; Franklin Lake, NJ, USA) according to the manufacturer's instructions. For MMP-9, TIMP-1, and TIMP-2, plasma was diluted 1:100, and this dilution was included in the final results.

### 2.5. Statistical Analysis

Statistical analyses were performed using SAS statistical software (SAS version 9.4; SAS Institute Inc., Cary, NC, USA). All continuous variables were tested for normal distribution using the Kolmogorov–Smirnov test. Descriptive statistics included medians with interquartile ranges (IQRs) for continuous variables that did not meet standard distribution criteria and proportions for categorical variables. Spearman rank correlation was used to examine associations between variables. Differences in continuous variables between the study and control groups were assessed using the Mann–Whitney *U* test; *p* values < 0.05 were considered statistically significant.

## 3. Results

### 3.1. Decreased Population of Total Regulatory T Cells But Increased Subsets of Functionally Active Regulatoty T Cells Expressing Latency-Associated Peptide in the Peripheral Blood of Biliary Atresia Patients

There were no changes in distribution pattern of the Th1 (CXCR3+), Th2 (CCR4+), Th17 (CCR6+), as well as Th17.1 (CXCR3+CCR6+) bearing CD4+ T lymphocyte subsets. The BA children showed slight but significant decrease in frequencies of total Treg cells but increase in numbers of Treg subsets expressing membrane latent TGF-*β* (LAP), but not GARP (LAP+GARP−), and activated Tregs bearing LAP+GARP+ phenotype (*p*=0.036, and *p*=0.032, respectively) (Table 4). Frequency of the Th17.1-bearing lymphocytes correlated positively with serum levels of the liver injury markers: AST and PT/INR (*P*=0.029; *r* = 0.514 and *p*=0.040; *r* = 0.486, respectively). The percentage of activated LAP+GARP+ Tregs was negatively correlated with serum Hb concentrations (*p*=0.009; *r* = -0.531).

### 3.2. The Biliary Atresia Children Had No Changes in the Peripheral B Cell Subsets Distribution Pattern

There were no changes in frequencies of the peripheral blood B cells, their naïve (CD27-) and memory (CD27+) counterparts, transitional and CD24high CD27+ B cell subsets and plasmablasts (Table 5, Figure S1). However, there were positive correlations between frequencies of the “naïve” B cells and serum ALT levels (*p*=0.037; *r* = 0.437) and of the “memory” B cells and serum GGT levels (*p*=0.002; *r* = 0.592).

### 3.3. The Bilary Atresia Children Peripheral Lymphocytes Had Increased Expression of Metalloproteinase-2 and Metalloproteinase-9

An increase in the frequency of lymphocytes expressing MMP-2 and MMP-9 was observed (*p* < 0.001, Table 6). A trends toward a lower density (MFI) of MMP-9 in neutrophils (*p*=0.081) and an increased density of MMP-9 in monocytes (*p*=0.056) were demonstrated.

### 3.4. Elevation of the Expression Levels of Metalloproteinase-9, Tissue Inhibitor of Matrix Metalloproteinase-1, Tissue Inhibitor of Matrix Metalloproteinase-2, Interleukin-6, Transforming Growth Factor-Beta, and Decreased Expression of Interleukin-21 and T-Box Transcription Factor Expressed in T Cells in the Peripheral Leukocytes of the Biliary Atresia Children

The BA children leukocytes had higher expression of *MMP-9* (*p* < 0.0001), *TIMP-1* (*p*=0.028), *TIMP-2* (*p*=0.007), *IL-6* (*p*=0.016), and *TGF-β* (*p*=0.023) than the leukocytes of the control group. Expression of the other genes tested was unchanged except for slightly decreased expression of *IL-21* (*p*=0.007) and *T-bet* (*p*=0.008) (Table 7, Figure S6A).

### 3.5. Correlations Between the Leukocyte Gene Expression Patterns and Some Serum Liver Injury Parameters of the Biliary Atresia Children

Leukocyte *MMP-2* expression correlated with *IL-17* (*p* < 0.001; *r* = 0.975), and *IL-21* (*p* < 0.001; *r* = 0.818). *TIMP-1* expression strongly correlated with *IL-6* (*p* < 0.001; *r* = 0.823), *CD161* (*p* < 0.001; *r* = 0.812), and *IL-1β* (*p*=0.005; *r* = 0.555). *IL-6* expression correlated with *CD161* (*p* < 0.001; *r* = 0.777), and *IL-1Ra* (*p*=0.001; *r* = 0.663), while *IL-17* correlated with *IL-21* (*p* < 0.001; *r* = 0.830). Expression of *FoxP3* correlated with *TGF-β* (*p*=0.001; *r* = 0.619), *GATA3* (*p*=0.001; *r* = 0.675) and *RORγT* (*p*=0.025; *r* = 0.458).

There were significant positive correlations between leukocyte inflammatory gene expression patterns and serum liver injury parameters: *FoxP3* correlated with AST (*p*=0.026; *r* = 0.453); *TIMP-1* and *IL-6* with GGT (*p*=0.004; *r* = 0.565; *p*=0.525, respectively), while *MMP-9* with total Bil (*p*=0.027, *r* = 0.452) and PT/INR (*p*=0.041; *r* = 0.419).

### 3.6. The Biliary Atresia Children Had High Tissue Inhibitor of Matrix Metalloproteinase-1 Plasma Levels and Significant Correlation Between Plasma Level Concentration of Selected Proteins and Leukocyte Gene Expression Profiles

The BA patients had almost three times higher plasma TIMP-1 levels than the control group (*p* < 0.001) (281.5 ng/ml vs 89.5 ng/ml). The levels of the other plasma proteins tested (IL-6, IL-17 A, MMP-9 and TIMP-2) were not altered (Table 8).

Plasma IL-6 levels correlated with direct Bil (*p*=0.010; *r* = 0.559), ALT (*p*=0.030; *r* = 0.487), and IL-17A (*p*=0.005; *r* = 0.600), MMP-9 correlated with TIMP-1 level (*p*=0.001; *r* = 0.678), and TIMP-1 correlated with TIMP-2 level (*p*=0.020; *r* = 0.516). There were also correlations between plasma inflammatory protein concentrations and leukocyte mRNA expression levels: MMP-9 plasma levels correlated with *IL-6* (*p*=0.008; *r* = 0.575) and *IL-1Ra* (*p*=0.004; *r* = 0.616); TIMP-1 levels with *IL-1Ra* (*p*=0.004; *r* = 0.615); and TIMP-2 levels with *CD161* mRNA expression (*p*=0.017; *r* = 0.528) (Table 9).

### 3.7. Increased Liver Expression of mRNA for Fork-Head Winged Helix Transcription Factor P3 and Tissue Inhibitor of Matrix Metalloproteinase-1 and Decline in the Liver Expression of Interleukin-1 Beta and Matrix Metalloproteinase-2 in the Biliary Atresia Children as Compared to the Control Group

There were no significant changes in the expression pattern of the most tested BA liver tissue genes as compared to the control group consisting of patients with cholestasis of unknown origin (*n* = 5) and bile duct cyst (*n* = 6). However, the expression of *FoxP3* and *TIMP-1* was 3-fold and twofold higher, respectively, in the BA patients as compared to the control group. Other significant changes included decreased expression of *IL-1β* and *MMP-2* (Table 10, Figure S6B).

### 3.8. Correlations Between Serum Liver Injury Indices and Liver Immune Markers

Liver *IL-6* and *TIMP-2* expression correlated with serum direct Bil levels (*p*=0.014; *r* = 0.554, and *p*=0.042; *r* = 0.498, respectively). Liver *TIMP-2* expression and serum ALT levels correlated with total Bil concentrations (*p*=0.034; *r* = 0.515; *p*=0.047; *r* = 0.488, respectively). Negative correlations included liver *FoxP3* expression vs. serum PT/INR levels (*r* = −0.538; *p*=0.018).

### 3.9. Mutual Correlations Between the Biliary Atresia Liver Gene Expression Pattern and Serum Inflammatory Proteins Levels

Liver ***TIMP-1*** expression correlated with liver *TIMP-2* (*r* = 0.854, *p*=0.0001), *MMP-12* (*r* = 0.591, *p*=0.015), *T bet* (*r* = 0.627, *p*=0.005), *CD161* (*r* = 0.726, *p*=0.0004), and serum IL-17 levels (*r* = 0.821, *p*=0.023). Liver ***TIMP****-2* expression correlated with *T bet* (*r* = 0.728, *p*=0.0009), *CD161* (*r* = 0.771, *p*=0.0003), and serum IL-17 levels (*r* = 0.857, *p*=0.013). Liver ***MMP-2*** expression correlated with liver *TIMP-1* (*r* = 0.542, *p*=0.017), *TIMP-2* (*r* = 0.546, *p*=0.023), and *MMP-12* (*r* = 0.698, *p*=0.002). **Liver *IL-17*** expression correlated with liver *IL-1 b* (*r* = 0.743, *p*=0.0003) as well as with liver *IL-21* (*r* = 0.901, *p*=0.0001), *MMP-2* (*r* = 0.818, *p*=0.0001), *MMP-9* (*r* = 0.510, *p*=0.043), and *MMP-12* (*r* = 0.665, *p*=0.005). Finally, liver ***IL-6*** expression correlated with liver *IL-1 beta* (*r* = 0.484, *p*=0.035) and *TIMP-1* (*r* = 0.51, *p*=0.024).

## 4. Discussion

The study aimed to identify leukocyte and liver immune markers whose expression would eventually be associated with the serum liver injury parameters AST, ALT, GGT, Bil (total and direct), Hb, and PT/INR. We found that the BA children had slight but significant decreased frequencies the total Treg cells but increased proportion of the activated Treg cells bearing LAP, and increased frequency of lymphocytes expressing MMP-2 and MMP-9. Leukocyte gene expression profile revealed increased expression of *TGF-β*, *IL-6*, *MMP-9*, *TIMP-1*, and *TIMP-2* but decreased expression of *IL-21* and *T-bet*. Liver tissue from the BA patients expressed high levels of *FoxP3* and increased expression of *TIMP-1* but decreased expression of *IL-1β* and *MMP-2* with no significant changes in the expression of other genes tested.

The percentage of LAP expressing Treg correlated negatively with Hb levels but positively with Bil (total and direct) and PT/INR levels. Notably, the frequency of Th17.1-bearing cells correlated with serum levels of ALT, AST, and PT/INR; the percentage of memory B cells correlated with GGT serum levels, and the percentage of naive B cells correlated positively with ALT levels. Regarding the leukocyte gene expression profiles, high expression of MMP-9 (more than 3.5-fold) positively correlated with total Bil and PT/INR and tended to be associated with increased ALT levels.

It has previously been found that during the first 90 days after HPE, activated NK cells, high IL-8, neutrophil by-products, and decreased Treg population directly correlated with poor outcomes based on increasing Bil and/or need for liver transplantation [5]. Our study did not find any correlation between frequency of total peripheral Treg cells and clinical outcomes. However, other studies in patients with BA and the rotavirus-induced mouse model of BA (murine BA) have shown a decreased number and function of Tregs [13–15]. It was also found that decreased peripheral blood Tregs frequency in the BA patients was associated with a lack of sufficient cytotoxic T lymphocyte-associated antigen-4 (CTLA-4) upregulation, which is necessary for cell-to-cell contact inhibition of inflammatory responses [16]. Found here increased proportion of activated Tregs expressing the LAP molecule and the strong negative association of the frequency of these cells with liver functional parameters (here Hb levels) may further point to the role of Tregs in controlling liver function during BA.

LAP-bearing Tregs represent a novel subset of T lymphocytes. The suppressive effect of CD4+LAP+T cells is dependent on TGF-*β*, IL-10, and cell-to-cell contact [17]. CD4+LAP+T cells are highly active and protect against autoimmunity in experimental models of encephalomyelitis, systemic lupus erythematosus, colitis, and diabetes [18–21]. The role of these cells in BA is unknown. It is possible that the deficit of classical Tregs often observed in BA is somehow replaced by another type of regulatory cells, such as activated CD4+LAP+GARP+T cells described here. We found increased leukocyte *TGF-β* expression in the BA patients, which was highly correlated with leukocyte *FoxP3* and *GATA-3* gene expression. This phenomenon may be due to increased TGF-*β* production by activated Tregs or Th2 lymphocytes or induction of *FoxP3* and *GATA-3* expression by TGF-*β*. TGF-*β* is a suppressive cytokine produced by Tregs and other cell types such as monocytes, macrophages and B cells [22, 23]. It also exerts profibrotic effects and its expression is associated with liver fibrosis [24]. However, despite the increased expression of leukocyte *TGF-β*, there were no correlations between its expression in leukocytes and the serum liver injury parameters concentrations. However, leukocyte cell derived chemotaxin 2 (LECT2) has recently been proposed as a potential diagnostic biomarker of BA. LECT2 expression in serum and liver macrophages was associated with increased TGF-beta 1 secretion by liver CD163 (+) M2 macrophages and liver fibrosis. TGF-beta1 upregulated the expression of LECT2 expression that correlated with liver fibrosis in the BA patients [25]. Found here positive correlations between numbers of peripheral naive and memory B cells with liver injury parameters (ALT and GGT serum levels, respectively) point out the known role of B cells in the BA liver pathology reflected by increased presence of intra-hepatic periductal B cells, IgG and IgM deposits, high levels of high affinity pathogenic IgG antibodies, and production of pro-inflammatory mediators that activate T cells, which exert liver injury by releasing cytokines and cytotoxic granules to attack cholangiocytes [26–29].

We observed that increased leukocyte *IL-6* expression (2.7-fold) strongly correlated with *CD161* and *IL-1b* expression. *CD161*-expressing leukocytes contain IL-17-producing precursors [30], which upon activation with IL-6, IL-1b, and IL-23 [31] differentiate into IL-17 cells known to be involved in the proinflammatory patterns of BA leading to liver dysfunction and fibrosis [32, 33]. Under these conditions, the generation of Tregs is limited, which may ultimately explain the deficit in Tregs generation and function in BA patients [24]. Here, we found a strong correlation between leukocyte *IL-6* expression and GGT levels and a tendency to increase frequency of Th17.1 cell subset, which correlated with liver injury parameters such as AST and PT/INR. Th17.1 cells are a subset of highly proinflammatory T cells that produce IL-17 and IFN-g [33]. This T cell subset differs from “classical” Th17 cells, which are nonpathogenic and produce mainly IL-17 and IL-10 [34, 35]. However, the conditions that induce pathogenic Th17.1 cells in humans are incompletely defined and their role in the BA development remains unknown.

In this regard, we found here that decreased leukocyte *IL-21* expression correlated with direct serum Bil levels as well as with leukocyte *IL-17A* expression. IL-21-dependent signaling is essential in regulating the differentiation of pathogenic Th17.1 cells in humans. IL-21 inhibits Th1 differentiation and GM-CSF production, thus preventing the generation of pathogenic Th1/IL-17 effector cells [36, 37]. Reduced leukocyte *IL-21* expression may therefore result in enhanced differentiation of CD4+ T cells into highly proinflammatory effectors with a Th17.1 phenotype, which correlates with the aforementioned liver injury parameters (AST, PT/INR). Accordingly, we can also speculate that a highly positive correlation between *IL-17A* leukocyte expression and low *IL-21* expression may represent an increase in activation of Th17.1 bearing T cells and an increase in IL-17 production by these Th17/IFN-*γ* co-expressing effectors. This observation is further supported here by the positive correlation between the expression of *CD161* and *T-bet*, where T-bet is a transcription factor of Th1 cells responsible for IFN-*γ* expression and CD161 is a marker of Th17-producing precursors [30].

High expression of leukocyte *MMP-9*, *TIMP-1*, and *TIMP-2*, as well as positive correlations between leukocyte *MMP-9* vs. liver function parameters (Bil, ALT, PT/INR) and *TIMP-1* expression vs. serum GGT levels, suggest the role of peripheral leukocyte MMP/TIMP system in liver inflammatory responses. Liver-infiltrating leukocytes (monocytes, neutrophils, and lymphocytes) participate in tissue repair and fibrosis. The main cause of liver fibrosis is a persistent new formation of ECM. ECM components are degraded by MMPs, which, together with their inhibitors (TIMPs), play a key role in maintaining the balance between fibrolysis and fibrogenesis [38]. As mentioned above, increased expression of leukocyte *MMP-9* and *TIMP-1* correlated with serum Bil, PT/INR, and GGT levels. In addition, increased leukocyte *TIMP-1* correlated with leukocyte *IL-6*, *IL-1β* and *CD161* expression. This suggests that leukocyte *TIMP-1* upregulation may be related to the action of Th17-inducing cytokines (*IL-1β*, *IL-6*), which in turn may induce the expression of *CD161*, a marker of Th17-producing precursors [30]. Upregulation of TIMP-1 may decrease MMP activities, which in turn promote fibrosis. TIMP-1 is a major marker of liver fibrosis [39].

At least twofold increase in *FoxP3* and *TIMP-1* expression in the BA liver confirms the known involvement of these molecules in BA pathology. It should be noted that the control group used here to estimate the gene expression pattern in the BA liver had a proinflammatory background because it was not possible to get liver samples from healthy age-matched children. It consisted of the subjects with cholestasis of unknown etiology (*n* = 5) and biliary cyst (*n* = 6). Nevertheless, the expression levels of *FoxP3* and *TIMP-1* still remained high in the BA liver, suggesting that the expression of these markers may be specifically related to the course of BA over the other inflammatory liver diseases. In addition, there was a negative correlation between BA liver *FoxP3* expression and serum PT/INR levels, indicating the possible protective role of BA liver *FoxP3* expression in the course of the disease. Here we also found that the BA liver *IL-17* expression strongly correlated with liver expression of *IL-1 beta*, *IL-21*, *MMP-2*, *MMP-9*, and *MMP-12*. IL-1 beta and IL-21 are known to be involved in the enhancement of IL-17 production and IL-17 may upregulate the expression of MMP-2, MMP-9, and MMP-12. We also showed that BA liver *IL-6* expression correlated with liver *IL-1 beta* as well as *TIMP-1* levels, and the BA liver *TIMP-1* expression (highly elevated) strongly correlated with *TIMP-2*, *MMP-12*, *T bet*, *CD161*, and IL-17 serum levels. Similarly, the BA liver *TIMP-2* correlated with *T bet*, *CD161*, and IL-17 serum levels. Finally, the BA liver *MMP-2* expression correlated with *TIMP-1*, *TIMP-2*, and *MMP-12* expression. Altogether these data suggest complex regulation of BA liver inflammatory pathways with known engagement of Th17/IL-17 axis in liver fibrosis. The Th17/IL-17 axis is known to be involved in fibrotic processes in the liver by activation of stellate cells, induction of TGF-beta, promotion of the myofibroblastic or epithelial–mesenchymal transition, and induction of imbalance between MMPs and TIMPs. Correlations presented here suggest the existence of complex ongoing inflammatory process in the BA liver that make difficult to find some specific biomarkers that would decisively distinguish the BA pathology from inflammatory other liver diseases [40–42]. Several other biomarkers have recently been proposed to differentiate BA from non BA including serum levels of MMP-7, interleukin 33, GGT, and to predict post-KPE liver fibrosis/cirrhosis by the use of the aspartate aminotransferase to platelet ratio index (APRi) and evaluation serum hyaluronic acid and MMP-7 levels [43].

There were some limitations of the study including: lack of cytometric analysis of lymphocytes from the liver (not liver tissue specimens of appropriate size available), and necessity to use the control group with inflammatory background for evaluation of the gene expression pattern in the BA liver. We did so because it was not possible to get liver samples from healthy age-matched children. It consisted of the subjects with cholestasis of unknown etiology (*n* = 5), and biliary cyst (*n* = 6). We realize that the group was small but we could not obtain more numerous controls simply because they were not available at the time of study performance. However, the statistical analysis (described in material and methods) made possible to present the results, including calculation of correlations between the tested samples (Spearman rank test), and to evaluate differences between the continuous variables and control groups, which were assessed by the Mann–Whitney *U* test, with *p* values <0.05 considered as statistically significant.

## 5. Conclusions

No predominant single markers in leukocytes and liver were uniquely associated with serum liver injury parameters. More complexed molecular studies are needed to find markers definitely related to BA including establishment of the immune-related genetic models that could distinguish BA from other cholestasis diseases [44].

## Figures and Tables

**Table 1:** Age and selected clinical parameters of the children with biliary atresia and control children.

	BA children *n* = 25	Control children *n* = 12	*p*-Value
	Mean ± SD	Mean ± SD	
Age (days)	82.55 ± 44.90	109.55 ± 6.31	n.s.
Age (months)	2.75 ± 1.50	3.66 ± 2.04	n.s.
Hb (g/dL)	37.07 ± 4.53	36.70 ± 4.64	n.s.
Bil total (mg/dL)	8.73 ± 3.91	1.88 ± 2.66	0.0001*⁣*^*∗*^
Bil direct (mg/dL)	7.23 ± 2.86	1.29 ± 2.35	0.0001*⁣*^*∗*^
ALT (IU/L)	141.24 ± 87.00	29.55 ± 21.16	0.0001*⁣*^*∗*^
AST (IU/L)	232.10 ± 191.75	52.85 ± 59.01	0.0001*⁣*^*∗*^
GGT (IU/L)	586.03 ± 412.44	132.55 ± 323.06	0.0001*⁣*^*∗*^
PT (INR)	1.08 ± 0.12	1.06 ± 0.08	n.s.

*⁣* ^*∗*^ *p* < 0.05, statistically significant differences.

**Table 2:** Fluorochrome-labeled monoclonal antibodies used in cytometry studies.

B cell subsets (naïve, memory, CD24highCD27+, transitional B cells and plasmablasts)	anti-CD19 FITC (clone 4G7), anti-CD24 PE (clone ML5-RUO), anti-CD27 PE-Cy7 (clone M-T271) and anti-CD38 BV421 (clone HIT2) (BD Biosciences).

Regulatory and recent thymic emigrant T cells	anti-CD4 APC-Cy7 (RPA-T4 clone), anti-CD25 BV421 (M-A251 clone), anti-CD127 PE or PerCP-Cy5.5 (HIL-7R-M21 clone), anti-CD45RO FITC (clone UCHL1), anti-CD45RA V500 (clone HI100), anti-latency-associated peptide (LAP) PerCP-Cy5.5 (clone TW4-2F8) and anti-glycoprotein A repetitions predominant (GARP) APC (clone 7B11), CD31 PE-Cy7 (clone WM59) (BD Biosciences), CD161 PE (clone 191B8) (Beckman Coulter)

Th subtypes (Th1, Th2, Th17 and Th17.1 cells)	anti-CD4 APC-Cy7 (RPA-T4 clone), anti-CD45RA V500 (clone HI100), CD45RO PE-Cy7 (clone UCHL1), CCR6 PerCP-Cy5.5 (clone 11A9), CXCR3 AF488 (clone 1C6/CXCR3), CCR4 BV 421 (clone 1G1) (BD Biosciences), CD161 PE (clone 191B8) (Beckman Coulter)

Leukocytes MMPs expression	anti-CD45 BV421 (clone HI30), (BD Bioscience), intracellular: anti-MMP-9 FITC (clone 56129), anti-MMP-2 PE (clone 1A10) or isotype control: IgG2b FITC (clone 133303), IgG2a PE (clone 20102) (RnD Systems)

**Table 3:** Primer sequences of target genes and reference gene for SYBR Green real-time PCR.

Gene	Forward primer	Reverse primer
*RORγT*	CTG GGC ATG TCC CGA GAT G	GAG GGG TCT TGA CCA CTG G
*FoxP3*	GTG GCC CGG ATG TGA GAA G	GGA GCC CTT GTC GGA TGA TG
*TGF-β*	GGA AAC CCA CAA CGA AAT CTA TG	CGG GTT CAG GTA CCG CTT C
*IL-17A*	CAA TCC CAC GAA ATC CAG GAT G	GGT GGA GAT TCC AAG GTG AGG
*IL-6*	TGA AAG CAG CAA AGA GGC ACT	GGC AAG TCT CCT CAT TGA ATC C
*IL-1β*	TTG TTG AGC CAG GCC TCT CT	ACC AAA TGT GGC CGT GG TT
*IL-21*	GTC ATC TGT CTG ATG GTC ATC TTC TT	TCA GGG ACC AAG TCA TTC ACA TA
*IL-1Ra*	GAA GAT GTG CCT GTC CTG TGT	CGC TCA GGT CAG TGA TGT TAA
*MMP-2*	TGA TCT TGA CCA GAA TAC CAT CGA	GGC TTG CGA GGG AAG AAG TT
*MMP-9*	CAA CAT CAC CTA TTG GAT CC	CGG GTG TAG AGT CTC TCG CT
*TIMP-1*	CTT CTG GCA TCC TGT TGT TG	AGA AGG CCG TCT GTG GGT
*TIMP-2*	CGA CAT TTA TGG CAA CCC TAT CA	CAG GCC CTT TGA ACA TCT TTA TCT
*MMP-12* (*only liver*)	F: TTC CCC TGA ACA GCT CTA CAA GCC TGG AAA	R: GAT CCA GGT CCA AAA GCA TGG GCT AGG ATT
*T-bet*	CGG GAG AAC TTT GAG TCC AT	CTG GGA ACA GGA TAC TGG TTG
*GATA3*	GGC TCT ACT ACA AGC TTC ACA	CGG GTT AAA CGA GCT GTT CT
*CD161*	AAA TGC AGT GTG GAC ATT CAA	CTC GGA GTT GCT GCC AAT A
*G3PDH*	GCG GGG CTC TCC AGA ACA TCA T	CCA GCC CCA GCG TCA AAG GTG

Abbreviations: CD161, C-type lectin; FoxP3, fork-head winged helix transcription factor P3; G3PDH, glyceraldehyde-3-phosphate dehydrogenase; GATA3, GATA-binding protein 3; IL- 17, -6, -1*β*, -21, interleukin 17, 6, 1*β*, or 21, respectively; IL-1 Ra, interleukin 1 receptor antagonist; MMP-2, -9, -12, metalloproteinase 2, 9 or 12; ROR*γ*T, retinoic acid receptor-related orphan nuclear receptor gamma-T; T-bet, T-box transcription factor expressed in T cells, also called TBX21; TGF-*β*, transforming growth factor beta; TIMP-1, -2, tissue inhibitor of matrix metalloproteinase 1 or 2.

**Table 4:** The distribution of peripheral blood regulatory T cells and their subsets in the BA children and controls.

		BA children	Control children	*p*-Value
		Median [IQR]	Median [IQR]	
Total Tregs	%	6.85 [5.79; 7.75]	7.76 [7.23; 8.16]	0.093
Naïve Tregs	%	84.91 [79.12; 88.03]	79.95 [77.92; 84.99]	0.391
Memory Tregs	%	12.00 [9.54; 18.22]	17.95 [11.77; 19.28]	0.365
GARP^+^LAP^−^ Tregs	%	0.96 [0.20; 2.58]	0.65 [0.08; 2.24]	0.704
GARP^−^LAP^+^ Tregs	%	2.36 [1.23; 4.00]	0.77 [0.00; 1.85]	0.036*⁣*^*∗*^
Activated Tregs	%	0.05 [0.00; 0.50]	0.00 [0.00; 0.00]	0.032*⁣*^*∗*^
Proinflammatory Tregs	%	8.28 [7.83; 16.51]	12.07 [7.03; 16.70]	0.915

*Note:* BA children: *n* = 23 (for proinflammatory Tregs: *n* = 7); control children: *n* = 12 (for proinflammatory Tregs: *n* = 9).

*⁣* ^*∗*^ *p* < 0.05, statistically significant differences.

**Table 5:** The distribution of peripheral blood B cell subsets in the BA children and controls.

		BA children	Control children	*p*-Value
		Median [IQR]	Median [IQR]	
Total B cells	%	12.30 [9.66; 16.20]	11.10 [7.18; 12.50]	0.204
Naïve B cells	%	71.84 [62.80; 79.17]	68.45 [61.43; 73.16]	0.121
Memory B cells	%	2.23 [1.53; 2.75]	2.40 [1.30; 4.46]	0.400
Transitional B cells	%	10.80 [7.33; 23.50]	15.70 [7.73; 18.40]	0.947
CD24^hi^CD27^+^B cells	%	6.31 [5.28; 9.01]	3.70 [1.49; 8.27]	0.129
Plasmablasts	%	1.15 [0.59; 1.83]	0.81 [0.45; 2.68]	0.792

*Note:* BA children: *n* = 23; control children: *n* = 12.

**Table 6:** Peripheral blood lymphocyte expression of MMP-2 and MMP-9 in the BA and control children.

Lymphocytes		BA children	Control children	*p*-Value
		Median [IQR]	Median [IQR]	
MMP-2	%	0.32 [0.17; 0.43]	0.10 [0.03; 0.19]	<0.001*⁣*^*∗*^
	MFI	651 [592; 727]	611 [579.50; 1148.50]	0.866

MMP-9	%	1.36 [0.98; 1.98]	0.34 [0.22; 0.60]	<0.001*⁣*^*∗*^
	MFI	1315 [1239; 1479]	1283 [1113.50; 1680.50]	0.613

*Note:* BA children, *n* = 21; control children, *n* = 12.

*⁣* ^*∗*^ *p* < 0.05, statistically significant differences.

**Table 7:** Leukocyte gene expression patterns of the BA and control children.

	BA children	Control children	*p*-Value
	Median [IQR]	Median [IQR]	
*MMP-2*	1.60 [0.66–7.10]	1.03 [0.98–1.04]	0.111
*MMP-9*	3.52 [1.44–6.46]	1.03 [0.98–1.04]	<0.0001*⁣*^*∗*^
*TIMP-1*	1.76 [1.00–2.23]	1.03 [0.98–1.04]	0.028*⁣*^*∗*^
*TIMP-2*	1.82 [1.09–2.38]	1.03 [0.98–1.04]	0.007*⁣*^*∗*^
*IL-6*	2.67 [1.00–5.28]	1.04 [0.99–1.05]	0.016*⁣*^*∗*^
*IL-1β*	1.52 [0.55–2.99]	1.04 [0.99–1.05]	0.582
*IL-17*	1.35 [0.56–5.70]	1.04 [0.99–1.05]	0.384
*IL-21*	0.60 [0.37–0.84]	1.03 [0.98–1.04]	0.007*⁣*^*∗*^
*IL-1Ra*	0.81 [0.51–1.07]	1.03 [0.98–1.04]	0.099
*TGFβ*	1.57 [0.97–1.89]	1.04 [0.99–1.05]	0.023*⁣*^*∗*^
*T-bet*	0.70 [0.33–0.96]	1.02 [0.97–1.03]	0.008*⁣*^*∗*^
*CD161*	1.09 [0.59–1.74]	1.02 [0.97–1.03]	0.840
*GATA3*	0.84 [0.54–1.46]	1.02 [0.97–1.03]	0.283
*FoxP3*	1.22 [0.75–2.03]	1.04 [0.99–1.05]	0.346
*RORγT*	0.89 [0.58–1.64]	1.04 [0.99–1.05]	0.499

*Note:* BA patients: *n* = 25; control children: *n* = 12.

*⁣* ^*∗*^ *p* < 0.05, statistically significant differences.

**Table 8:** Plasma levels of inflammatory mediators in the BA children and controls.

	*p*-Value	BA children	Control children
		Median [IQR]	Median [IQR]
IL-6 (pg/ml)	0.683	23.00 [12.33–44.68]	31.96 [12.97–82.41]
IL-17 (pg/ml)	0.699	183 [0.00–754.68]	167.00 [0.00–475.13]
MMP-9 (ng/ml)	0.892	18.40 [14.92–31.67]	22.00 [14.31–26.40]
TIMP-1 (ng/ml)	<0.0001	281.48 [262.33–316.18]	89.54 [83.86–111.93]
TIMP-2 (ng/ml)	0.460	101.72 [96.17–121.30]	101.94 [93.73–115.11]

Abbreviations: IL-6, -17, interleukin-6, -17, respectively; MMP-9, metalloproteinase 9; TIMP-1, -2, tissue inhibitor of matrix metalloproteinase-1 or -2.

**Table 9:** Spearman's rank correlation between plasma-level concentration of selected proteins and leukocyte gene expression profiles in children with biliary atresia.

	IL-17A ELISA (pg/ml)	TIMP-1 ELISA (ng/dl)	TIMP-2 ELISA (ng/dl)	*IL-6* mRNA expression	*IL-1Ra* mRNA expression	*IL-21* mRNA expression	*CD161* mRNA expression
IL-6 ELISA	*p*=0.005*⁣*^*∗*^ *r* = 0.600	—	—	—	—	—	—
MMP-9 ELISA	—	*p*=0.001*⁣*^*∗*^ *r* = 0.678	—	*p*=0.008*⁣*^*∗*^ *r* = 0.575	*p*=0.004*⁣*^*∗*^ *r* = 0.616	—	—
TIMP-1 ELISA	—	—	*p*=0.02*⁣*^*∗*^ *r* = 0.516	—	*p*=0.004*⁣*^*∗*^ *r* = 0.615	—	—
TIMP-2 ELISA	—	—	—	—	—	—	*p*=0.017*⁣*^*∗*^ *r* = 0.528

*⁣* ^*∗*^Significant correlations (*p* < 0.05) are presented.

**Table 10:** Liver tissue specimens gene expression profiles in the BA children and controls.

	BA patients Median [IQR]	Controls Median [IQR]	*p*-Value
*RORγT*	0.39 [0.30–1.16]	0.98 [0.91–1.17]	0.0641
*FoxP3*	3.12 [2.54–5.55]	1.00 [0.92–1.14]	**0.0043**
*TGFβ*	0.83 [0.45 −2.16]	0.99 [0.91–1.17]	0.2818
*IL-17*	1.00 [0.45–3.98]	0.98 [0.91–1.17]	0.7961
*IL-6*	1.95 [0.48–4.59]	0.99 [0.91–1.13]	0.3499
*IL-1β*	0.33 [0.17–0.89]	1.00 [0.88–1.14]	**0.0142**
*IL-21*	0.48 [0.33–2.19]	0.98 [0.91–1.17]	0.0850
*IL-1Ra*	1.00 [0.57–4.85]	1.00 [0.92–1.09]	0.9143
*MMP-2*	0.42 [0.29–1.11]	0.98 [0.93–1.12]	**0.0251**
*MMP-9*	1.20 [0.82–4.40]	1.04 [0.98–1.07]	0.4609
*TIMP-1*	2.15 [1.64–3.34]	0.97 [0.94–1.11]	**0.0001**
*TIMP-2*	0.92 [0.71–1.12]	0.97 [0.94–1.11]	0.4201
*MMP-12*	0.42 [0.25–1.02]	0.99 [0.93–1.10]	0.0648
*T-bet*	0.51 [0.28–1.74]	1.05 [0.91–1.09]	0.1460
*GATA3*	1.46 [0.53–2.33]	0.99 [0.94–1.05]	0.2801
*CD161*	0.71 [0.55–1.30]	0.96 [0.93–1.110]	0.1839

*Note:* BA patients: *n* = 19; control children: *n* = 11. Bold *p*-values, statistically significant differences (*p* < 0.05).

## Data Availability

All data contained in the manuscript are available upon request.

## Nomenclature

Hb: hemoglobin
Bil: bilirubin
ALT: alanine aminotransferase
AST: aspartate aminotransferase
GGT: gamma glutamyltransferase
PT/INR: international normalized ratio of prothrombin time
ROR*γ*T: retinoic acid receptor-related orphan nuclear receptor gamma
FoxP3: fork-head winged helix transcription factor P3
TGF-*β*: transforming growth factor beta
IL-17, -6, -1*β*, -21: interleukin-17, -6, -1*β*, -21, respectively
IL-Ra: interleukin 1 receptor antagonist
MMP-2, -9: metalloproteinase-2 or -9
TIMP-1, 2: tissue inhibitor of matrix metalloproteinase-1 or -2
T-bet: T-box transcription factor expressed in T cells, also called TBX21
GATA-3: GATA-binding protein 3
CD161: C-type lectin
GARP: glycoprotein-A repetition predominant protein
LAP: latency-associated peptide
MFI: median fluorescence intensity.

## Supporting Information

Supporting Information 1 Figure S1: The gating strategy and representative dot plots for flow cytometry analysis of peripheral B cells and their subsets. Gating strategies of peripheral B cells, naïve (CD27-), memory (CD27+), CD24hiCD27+, and transitional (CD24highCD38hi) B cells, and plasmablast (CD24lowCD38hi) in flow-cytometry analyses (1A). Representative dot plots of peripheral B cells and their subsets frequencies in BA and control group (1B).

Supporting Information 2 Figure S2: The gating strategy of peripheral T cell subsets: naïve, memory, and CD31+ recent thymic emigrant.

Supporting Information 3 Figure S3: The gating strategy and representative dot plots for flow cytometry analysis of peripheral Th cells and their subsets. The gating strategies of peripheral Th1 (CD161+/-CCR6-CXCR3+CCR4-), Th2 (CD161+/-CCR6-CXCR3-CCR4+), Th17 (CD161+CCR6+CXCR3+CCR4-), and Th17.1 (CD161+CCR6+CXCR3-CCR4+) cells in flow cytometry analyses.

Supporting Information 4 Figure S4: The gating strategy of peripheral Treg cells and their subsets: naïve, memory, LAP+GARP-, activated (LAP+GARP+) and LAP-GARP+ Tregs.

Supporting Information 5 Figure S5: The gating strategy of peripheral blood leukocyte expression of MMP-2 and MMP-9.

Supporting Information 6 Figure S6: (A): Leukocyte gene expression levels in children with BA; median IQR; significant difference (*p* < 0.5). (B) Liver tissue specimens gene expression levels in children with BA; median IQR; significant difference (*p* < 0.5).
