# Supplementary material for: Correlation of the Expression Profile of Peripheral Leukocyte and Liver Tissue Immune Markers With Serum Liver Injury Indices in Children With Biliary Atresia
Source: Mediators Inflamm. 2025 Apr 16;2025:9889239. doi: 10.1155/mi/9889239 (PMC12017958; doi:10.1155/mi/9889239)
Supplement: Supporting Information 1 — Figure S1: The gating strategy and representative dot plots for flow cytometry analysis of peripheral B cells and their subsets. Gating strategies of peripheral B cells, naïve (CD27-), memory (CD27+), CD24hiCD27+, and transitional (CD24highCD38hi) B cells, and plasmablast (CD24lowCD38hi) in flow-cytometry analyses (1A). Representative dot plots of peripheral B cells and their subsets frequencies in BA and control group (1B). [file 9889239.f1.docx]

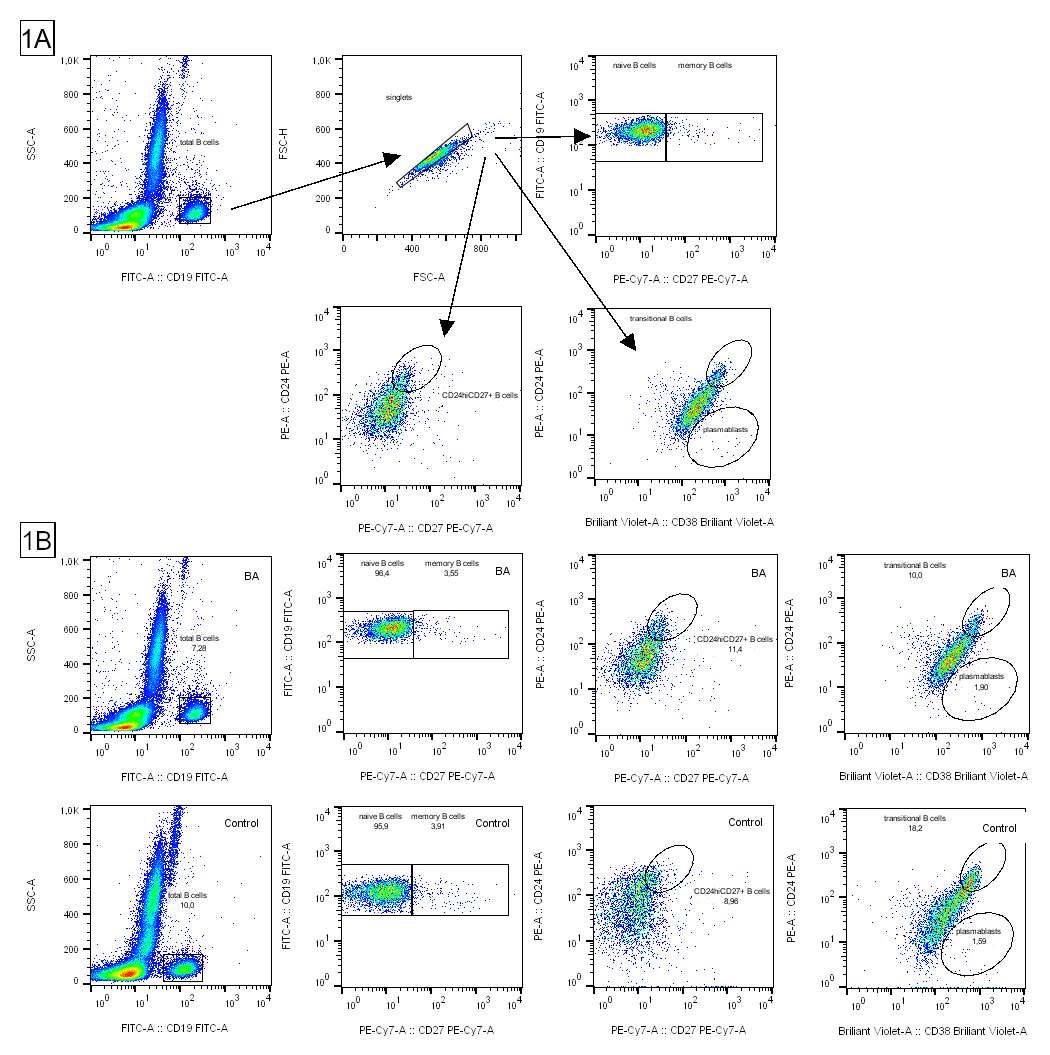


**Figure 1.** The gating strategy and representative dot plots for flow cytometry analysis of peripheral B cells and their subsets. Gating strategies of peripheral B cells, naïve (CD27-), memory (CD27+), CD24hiCD27+, and transitional (CD24highCD38hi) B cells, and plasmablast (CD24lowCD38hi) in flow-cytometry analyses (**1A**). Representative dot plots of peripheral B cells and their subsets frequencies in BA and control group (**1B**).
