# Supplementary material for: Correlation of the Expression Profile of Peripheral Leukocyte and Liver Tissue Immune Markers With Serum Liver Injury Indices in Children With Biliary Atresia
Source: Mediators Inflamm. 2025 Apr 16;2025:9889239. doi: 10.1155/mi/9889239 (PMC12017958; doi:10.1155/mi/9889239)
Supplement: Supporting Information 2 — Figure S2: The gating strategy of peripheral T cell subsets: naïve, memory, and CD31+ recent thymic emigrant. [file 9889239.f2.docx]

**
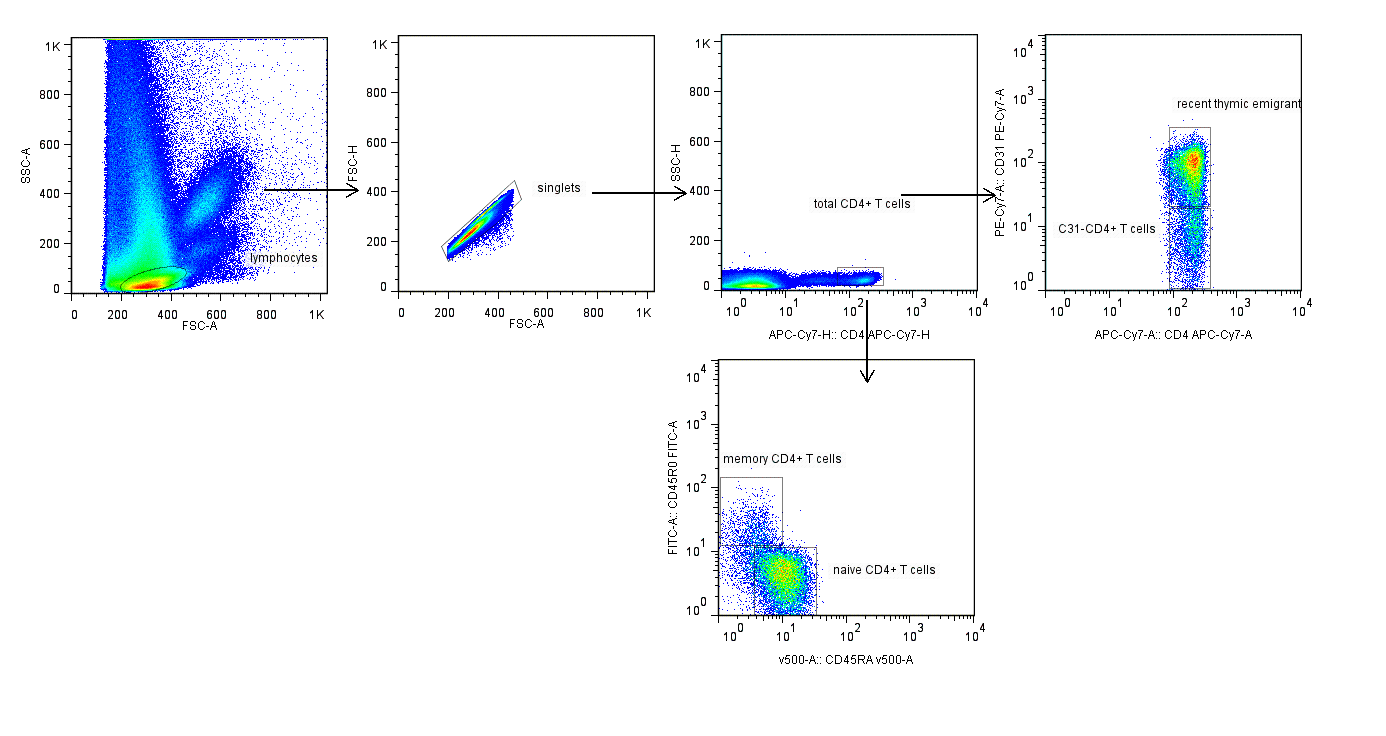
**

**Figure 2.** The gating strategy of peripheral T cell subsets: naïve, memory and CD31+ recent thymic emigrant.
