# Supplementary material for: Correlation of the Expression Profile of Peripheral Leukocyte and Liver Tissue Immune Markers With Serum Liver Injury Indices in Children With Biliary Atresia
Source: Mediators Inflamm. 2025 Apr 16;2025:9889239. doi: 10.1155/mi/9889239 (PMC12017958; doi:10.1155/mi/9889239)
Supplement: Supporting Information 3 — Figure S3: The gating strategy and representative dot plots for flow cytometry analysis of peripheral Th cells and their subsets. The gating strategies of peripheral Th1 (CD161+/-CCR6-CXCR3+CCR4-), Th2 (CD161+/-CCR6-CXCR3-CCR4+), Th17 (CD161+CCR6+CXCR3+CCR4-), and Th17.1 (CD161+CCR6+CXCR3-CCR4+) cells in flow cytometry analyses. [file 9889239.f3.docx]

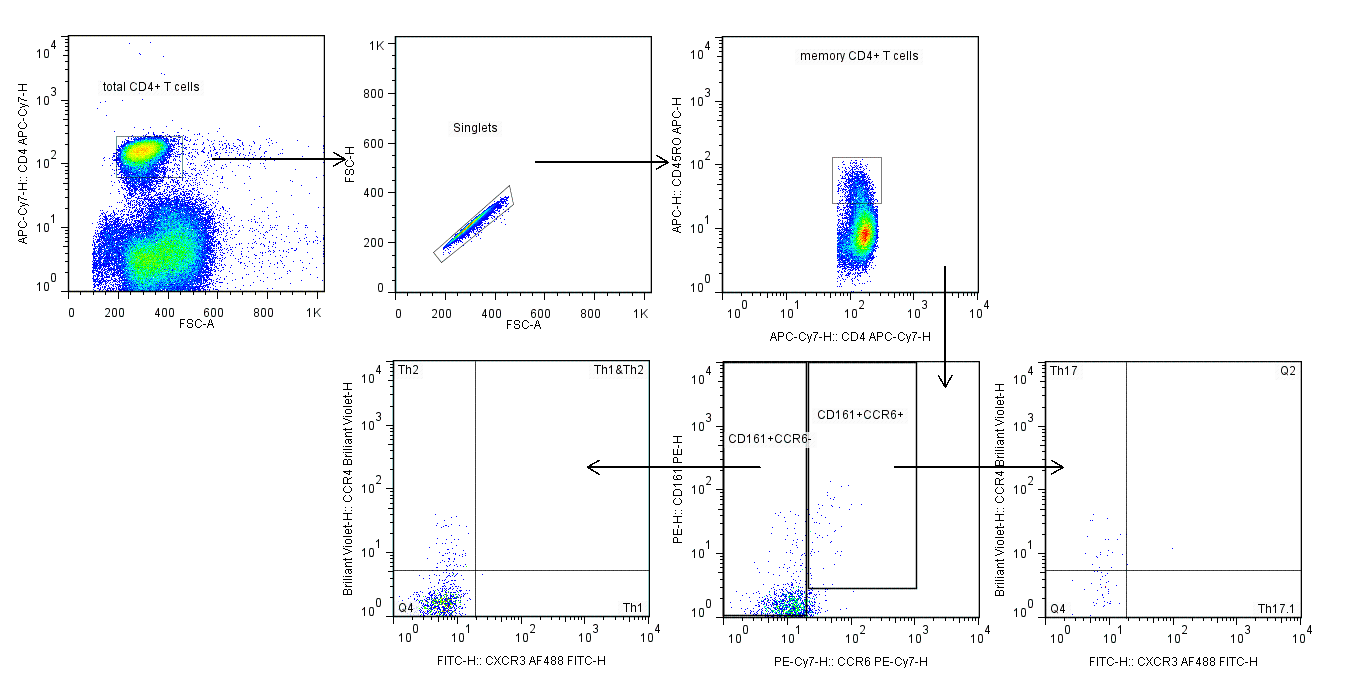


**Figure 3.** The gating strategy and representative dot plots for flow cytometry analysis of peripheral Th cells and their subsets. The gating strategies of peripheral Th1 (CD161+/-CCR6-CXCR3+CCR4-), Th2 (CD161+/-CCR6-CXCR3-CCR4+), Th17 (CD161+CCR6+CXCR3+CCR4-), and Th17.1 (CD161+CCR6+CXCR3-CCR4+) cells in flow cytometry analyses.
