# Supplementary material for: Correlation of the Expression Profile of Peripheral Leukocyte and Liver Tissue Immune Markers With Serum Liver Injury Indices in Children With Biliary Atresia
Source: Mediators Inflamm. 2025 Apr 16;2025:9889239. doi: 10.1155/mi/9889239 (PMC12017958; doi:10.1155/mi/9889239)
Supplement: Supporting Information 4 — Figure S4: The gating strategy of peripheral Treg cells and their subsets: naïve, memory, LAP+GARP-, activated (LAP+GARP+) and LAP-GARP+ Tregs. [file 9889239.f4.docx]

**
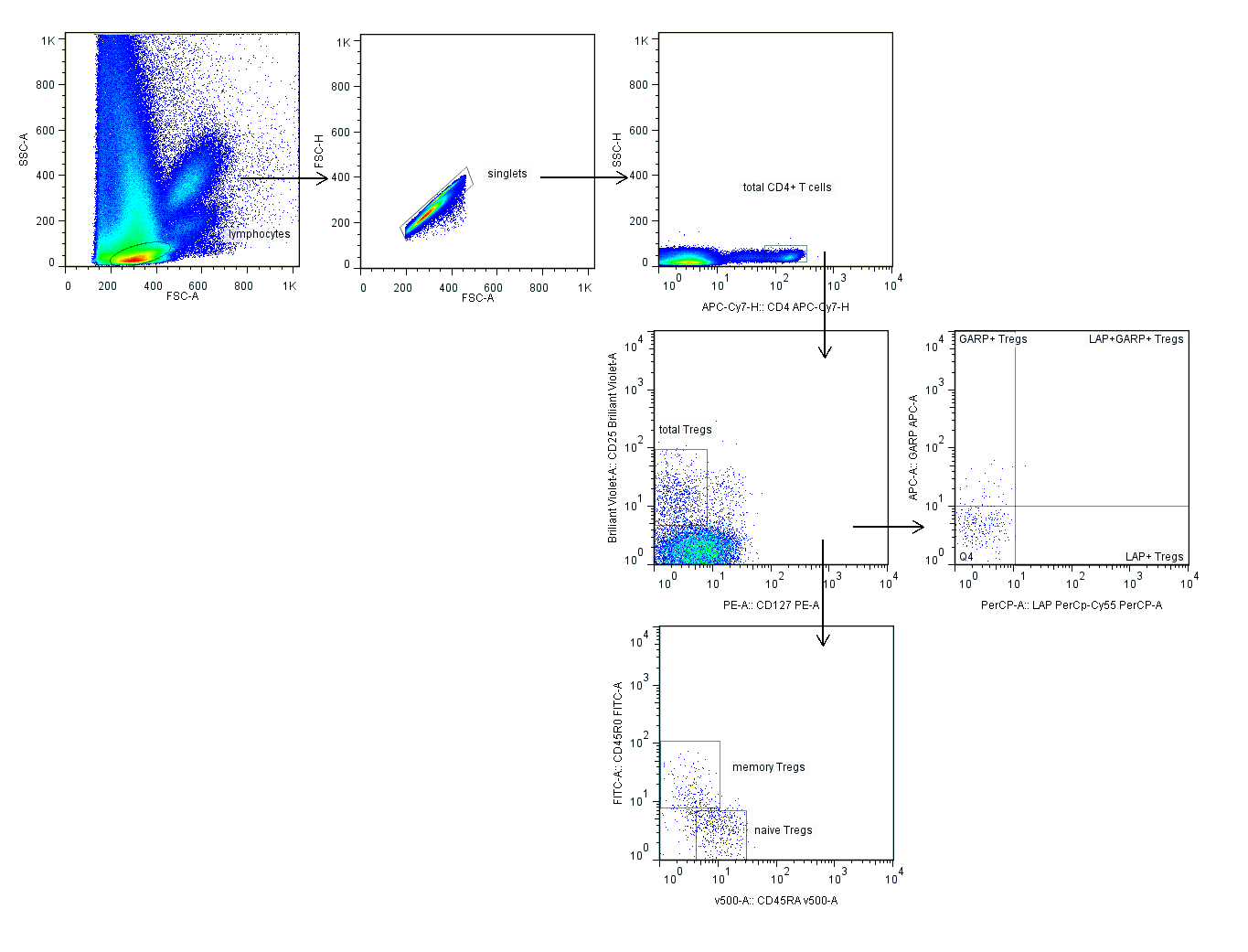
**

# Figure 4. The gating strategy of peripheral Treg cells and their subsets: naïve, memory, LAP+GARP-, activated (LAP+GARP+) and LAP-GARP+ Tregs.
