# Supplementary figures and images for: Correlation of the Expression Profile of Peripheral Leukocyte and Liver Tissue Immune Markers With Serum Liver Injury Indices in Children With Biliary Atresia
Source: Mediators Inflamm. 2025 Apr 16;2025:9889239. doi: 10.1155/mi/9889239 (PMC12017958; doi:10.1155/mi/9889239)

**
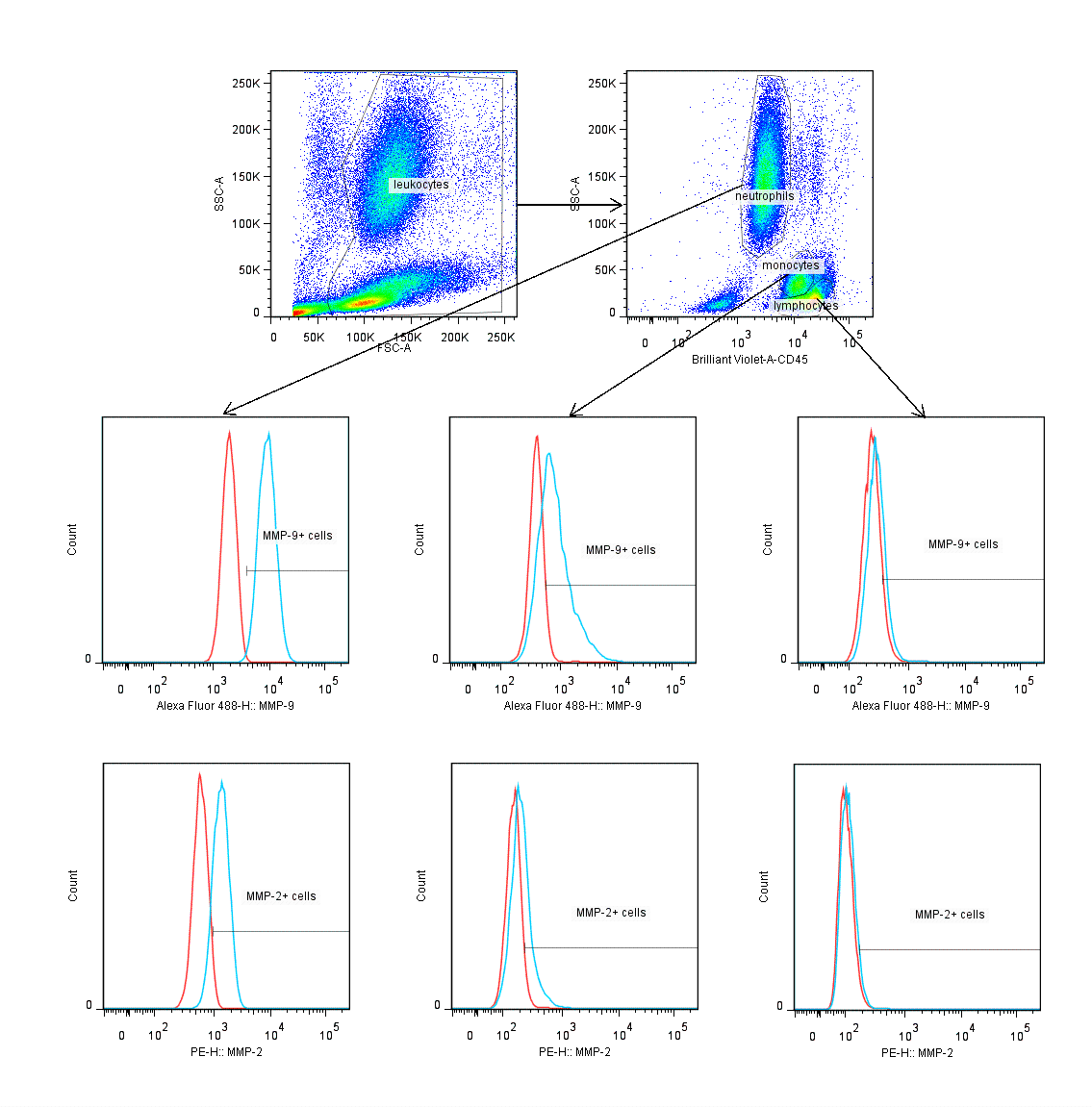
**

**Figure 5.** The gating strategy of peripheral blood leukocyte expression of MMP-2 and MMP-9.

Supplement: Supporting Information 5 — Figure S5: The gating strategy of peripheral blood leukocyte expression of MMP-2 and MMP-9. [file 9889239.f5.docx]
