# Supplementary material for: Correlation of the Expression Profile of Peripheral Leukocyte and Liver Tissue Immune Markers With Serum Liver Injury Indices in Children With Biliary Atresia
Source: Mediators Inflamm. 2025 Apr 16;2025:9889239. doi: 10.1155/mi/9889239 (PMC12017958; doi:10.1155/mi/9889239)
Supplement: Supporting Information 6 — Figure S6: (A): Leukocyte gene expression levels in children with BA; median IQR; significant difference (p < 0.5). (B) Liver tissue specimens gene expression levels in children with BA; median IQR; significant difference (p < 0.5). [file 9889239.f6.docx]

Fig. 6A Leukocyte gene expression levels in children with BA; median IQR; significant difference (p<0.5).

Fig. 6B Liver tissue specimens gene expression levels in children with BA; median IQR; significant difference (p<0.5)
